# Reliability and validity of the World Health Organization reading standards for paediatric chest radiographs used in the field in an impact study of Pneumococcal Conjugate Vaccine in Kilifi, Kenya

**DOI:** 10.1371/journal.pone.0200715

**Published:** 2018-07-25

**Authors:** M. Ominde, J. Sande, M. Ooko, C. Bottomley, R. Benamore, K. Park, J. Ignas, K. Maitland, T. Bwanaali, F. Gleeson, A. Scott

**Affiliations:** 1 KEMRI-Wellcome Trust Research Programme, Kilifi, Kenya; 2 Aga Khan University Hospital, Nairobi, Kenya; 3 Department of Infectious Disease Epidemiology, London School of Hygiene & Tropical Medicine, London, United Kingdom; 4 Oxford University Hospitals Foundation NHS Trust, Oxford, United Kingdom; 5 Imperial College, London, United Kingdom; 6 Oxford University, Oxford, United Kingdom; IAVI, UNITED STATES

## Abstract

**Background:**

Radiologically-confirmed pneumonia (RCP) is a specific end-point used in trials of Pneumococcal Conjugate Vaccine (PCV) to estimate vaccine efficacy. However, chest radiograph (CXR) interpretation varies within and between readers. We measured the repeatability and reliability of paediatric CXR interpretation using percent agreement and Cohen’s Kappa and the validity of field readings against expert review in a study of the impact of PCV on pneumonia.

**Methods:**

CXRs were obtained from 2716 children admitted between 2006 and 2014 to Kilifi County Hospital, Kilifi, Kenya, with clinically-defined severe or very-severe pneumonia. Five clinicians and radiologists attended a three-day training course on CXR interpretation using a WHO standard. All CXRs were read once by two local primary readers. Discordant readings and 13% of concordant readings were arbitrated by a panel of three expert radiologists. To assess repeatability, a 5% median random sample was presented twice. Sensitivity and specificity of the primary readers’ interpretations was estimated against the ‘gold-standard’ of the arbitrators’ results.

**Results:**

Of 2716 CXRs, 2 were uninterpretable and 159 were evaluated twice. The percent agreement and Kappa for RCP were 89% and 0.68 and ranged between 84–97% and 0.19–0.68, respectively, for all pathological findings. Intra-observer repeatability was similar to inter-observer reliability. Sensitivities of the primary readers to detect RCP were 69% and 73%; specificities were 96% and 95%.

**Conclusion:**

Intra- and inter-observer agreements on interpretations of radiologically-confirmed pneumonia are fair to good. Reasonable sensitivity and high specificity make radiologically-confirmed pneumonia, determined in the field, a suitable measure of relative vaccine effectiveness.

## Introduction

Acute respiratory tract infections (ARI) are responsible for almost 1 million childhood deaths annually[1]. Pneumococcal pneumonia is the leading bacterial aetiological agent and is the pathogen most frequently associated with fatal cases. Pneumococcal conjugate vaccines are highly efficacious against invasive pneumococcal disease but their effectiveness against pneumonia is low because the pneumococcus is not the only cause of pneumonia.

To evaluate the efficacy of Pneumococcal Conjugate Vaccine (PCV) against pneumonia, the World Health Organization (WHO) developed an interpretive standard for reporting chest radiographs (CXRs) of children with suspected pneumonia. By this standard, radiologically-confirmed pneumonia (RCP), also called primary end-point pneumonia, is defined as the presence of consolidation or pleural effusion or both. The WHO training consists of a library of 222 CXRs, accompanying software and instructions[2]. In addition, early adopters of the standard arranged joint review meetings to ensure consistency in the interpretation of the defined features. In a multi-centre reliability study, the decisions of 20 readers reviewing 100 out of the 222 reference radiographs demonstrated good agreement; 19 out of 20 readers had a Cohen’s Kappa index of >0.6 when compared to the reference standard of the majority conclusion[3].

The WHO standard was subsequently used in several vaccine trials; the effectiveness of different PCVs against RCP was estimated at 17%-37% suggesting that the end-point is relatively specific for pneumococcal pneumonia[4–8]. When designing the PCV10 Impact Study (PCVIS), a before-after vaccine effectiveness study based on a single hospital serving a well-defined population in Kilifi, Kenya[9], we chose to use the WHO interpretative standard[2]. All CXRs from children with pneumonia were read in the field by a radiologist and a medical officer. A panel of three experienced radiologists based in Oxford, UK, provided arbitration. To determine the validity of the readings in the field we compared the results of the primary readers against those of the arbitration panel on a sub-set of the CXRs within the study. To examine the repeatability and reliability of the primary readers we examined the intra- and inter-observer variation among radiological readings in the course of the impact study and compared the results with the primary reliability study undertaken by WHO[3].

## Methods

### Study population

The study population for PCVIS was defined by the Kilifi Health and Demographic Surveillance System (KHDSS), which comprises 280,000 residents living on the Indian Ocean Coast of Kenya[10]. The area is served by a single inpatient government facility, Kilifi County Hospital (KCH). All children aged ≥2 months to <12 years, admitted to Kilifi County Hospital between April 2006 and March 2014, were eligible for study.

Demographic and clinical data were captured using an existing electronic health information system at the point of admission. Children who had clinical features of severe or very severe pneumonia on admission to KCH were identified by an algorithm on the electronic data capture system and were recruited into the study. The clinical definitions were taken from WHO standards[11]. Severe pneumonia was defined as cough or difficulty breathing and in-drawing. Very severe pneumonia was defined as cough or difficulty breathing and at least one of the following danger signs; central cyanosis, inability to drink, prostrate, unconscious or head nodding.

CXRs were acquired within the first 48 hours of admission.

### Image acquisition

The parents or guardians of an eligible child were informed about the study and asked to consent to radiological investigation of their child. Each participant had a single erect antero-posterior, postero-anterior or supine antero-posterior CXR performed at the radiology department, depending on the child’s age and clinical status. Before March 2012, children who needed oxygen were transported to the department with a portable oxygen cylinder. After March 2012, CXRs from oxygen-dependent children were obtained using a portable machine on the wards. Images were immediately used for clinical management and were subsequently archived for analysis.

### Archiving

Prior to August 2011, radiographs were acquired as hard copy analogue films and later digitized using a ‘*Vidar Pro Advantage’* digitizer. The images were encoded using *Hipax* imaging software into DICOM images at 150dpi and 12-bit pixel depth. From August 2011 onwards, images were acquired using computed radiography (CR) and processed using a *Philips PCR Eleva S* laser reader. The cassette parameters were: 10 by 12 inches image area with a matrix size of 1670 by 2010 pixels. Owing to the large size of the DICOM images, they were compressed to lossy JPEG format, without significantly changing the spatial resolution, to optimize for distribution and reading. The CR images were anonymised prior to compression and scanned images were de-identified by cropping out hard-coded patient information. Identifiers on the images that could indicate the instrumental source of the radiograph were removed.

In December 2013, the CXRs were batched into clusters of 95. Each batch contained a similar proportion of post-vaccine radiographs. We retained a random sample of pre-vaccine radiographs for incorporation into subsequent batches of participants, yet to be recruited. Out of each batch of 95 images, 5 were randomly sampled and re-inserted into another batch to assess repeatability. The process resulted in batches of approximately 100 images each, as outlined in Table 1. These batches were numbered and read in order. Repeat images were inserted into a batch at least 4 batches distant from their original presentation, which meant that there was a time lag between the two reviews, to minimize recall bias. Within batches, the sequence of presentation was randomized.

**Table 1:** Distribution of 2,875 chest radiographs within the batches according to the vaccine period and repeat readings for intra-observer variability evaluation.

Batch No.	Pre-vaccine period images	Post-vaccine period images		Total images	Duplicate images	
	n	n	*%*	N	n	*%*
1	75	25	*25*	100	5	5
2	69	31	*31*	100	5	5
3	75	21	*22*	96	5	5
4	73	25	*26*	98	5	5
5	71	25	*26*	96	5	5
6	75	23	*23*	98	5	5
7	73	27	*27*	100	5	5
8	72	25	*26*	97	5	5
9	62	37	*37*	99	5	5
10	71	26	*27*	97	5	5
11	67	29	*30*	96	5	5
12	71	28	*28*	99	5	5
13	71	26	*27*	97	4	4
14	78	20	*20*	98	5	5
15	73	24	*25*	97	5	5
16	74	23	*24*	97	5	5
17	69	29	*30*	98	5	5
18	75	22	*23*	97	5	5
19	77	21	*21*	98	5	5
20	75	25	*25*	100	5	5
21	68	31	*31*	99	5	5
22	81	17	*17*	98	5	5
23	72	28	*28*	100	6	6
24	70	27	*28*	97	5	5
25	22	3	*12*	25	5	20
26	38	47	*55*	85	5	6
27	30	56	*65*	86	7	8
28	38	49	*56*	87	6	7
29	28	49	*64*	77	6	8
30	29	69	*70*	98	5	5
31	24	41	*63*	65	5	8
All	1946	929	*32*	2,875	159	*6*

### Applying the WHO interpretive standard

A medical officer at the KEMRI-Wellcome Trust Research Programme and a consultant radiologist, based in Nairobi, acted as primary readers. The arbitration panel consisted of three readers, all of whom were experienced consultant radiologists at Oxford University Hospitals NHS Foundation Trust in Oxford, UK. In August 2012, all five readers attended a two-day training course in London, on the WHO interpretive standards for paediatric CXRs, in conjunction with readers and trainers from another project, the Pneumonia Etiology for Child Health (PERCH) project[12]. One of the facilitators of this training session was a member of the original panel that defined the WHO standard. The objective of the training was to agree upon the implementation of the WHO standard and to calibrate interpretations to the majority view on the existing library of 222 paediatric CXRs[3].

The WHO standard requires readers to first classify radiographs into three grades of quality[2]: (i) Adequate/optimal: this indicates that the radiograph was adequate to draw a conclusion of consolidation, pleural effusion or Other Infiltrate. (ii) Sub-optimal: this indicates that there were one or more deficiencies such as over/under penetration, rotation, inadequate inspiration or inadequate field of view but it was still possible to determine whether the image contained consolidation and/or pleural effusion. In these images the radiological classification ‘Other Infiltrate’ could not be made. (iii) Uninterpretable: these were radiographs in which none of the three pathological endpoints could be identified.

The WHO standard next requires readers to identify pathological findings; three categories of finding are specified: (i) End-point consolidation, abbreviated hereafter as ‘consolidation’, is defined as a dense or fluffy opacity that occupies a portion or whole of a lobe or of the entire lung, that may or may not contain air-bronchograms. Atelectasis of an entire lobe that produces a dense opacity or a positive silhouette sign (loss of the visible margin of the diaphragmatic or cardiac shadow) were also considered as indicating consolidation. For opacities, we applied an arbitrary minimum size of a rib and an adjacent intercostal space. (ii) Pleural effusion is defined as fluid in the lateral pleural space between the lung and chest wall but not fluid confined to the horizontal or oblique fissures. (iii) Other Infiltrate is defined as linear and patchy densities in a lacy pattern, featuring peribronchial thickening and multiple areas of atelectasis. Miliary or mixed alveolar and interstitial shadows, patchy infiltrates that are not of sufficient size to constitute primary end-point consolidation, small areas of atelectasis and extension of bronchovascular markings into the lateral third or apical lung field are also included as Other Infiltrate. Apical extension only applied to erect CXRs[2].

Finally, the WHO standard requires readers to interpret the pattern of pathological findings, and again, three categories of conclusion are specified: (i) Primary end-point pneumonia, also described as Radiologically-confirmed pneumonia (RCP) [7] is defined by the presence of either consolidation or pleural effusion. To indicate RCP the pleural effusion must be in the lateral pleural space, not just in the minor or oblique fissure, and must be spatially associated with the area of consolidation/Other Infiltrate; alternatively, it must be of sufficient large volume as to potentially obscure an area of consolidation. (ii) Other Infiltrate is defined by identifying the pathological finding ‘Other Infiltrate’, above, in the absence of a pleural effusion. (iii) No consolidation/infiltrate/effusion describes the remainder[2].

### Reading radiographic images

We used viewing station/display specifications adopted from the American College of Radiology; 2.5–5 line-pairs/mm spatial resolution at a minimum 10-bit pixel depth[13]. A standardized SMPTE test pattern was applied prior to each reading session to aid in the calibration of the image-viewing monitors[14].

The images were read between January 2013 and October 2014. The two primary readers examined the images and recorded their interpretations independently. For each CXR, readers identified the three possible pathological findings of the WHO standard; consolidation, pleural effusion or Other Infiltrate; a reader could identify one, two or three of these end-points in each image. Given the context of a PCV impact study, our primary focus was on the conclusion RCP. Therefore, in examining the validity and reliability of radiological readings we first analysed the three pathological findings (consolidation, pleural effusion or Other Infiltrate) and then analysed the principal relevant conclusion (RCP).

**Fig 1:**
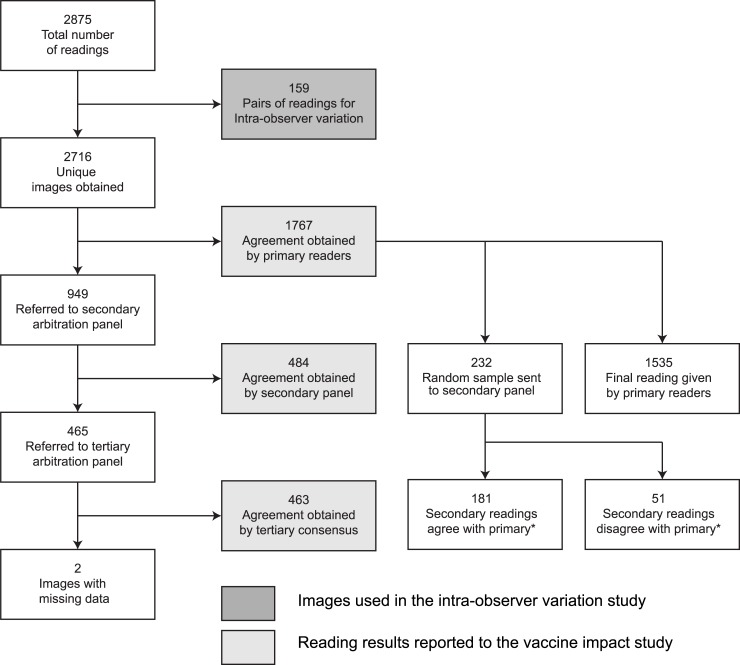
Flow chart showing selection of chest radiographs for the separate analyses. Fig 1 footnote: * Of 232 CXRs with agreement between the two primary readers, the arbitration panel agreed with the primary readers (on all radiological criteria) in 181 and there was at least one disagreement between primary readers and the arbitration panel in 51. For RCP, there was agreement between primary readers and the arbitration panel in 219; in 13 radiographs the arbitration panel found RCP but the primary readers did not.

All the images on which the primary readers disagreed, and a random sample of those on which they agreed, were sent to the arbitration panel. This was set at 10% but, because of an error in coding the project software, 13% of concordant images were selected. Two members of the arbitration panel (secondary arbitration) read these images independently of each other in a second level of review. In situations where these two arbiters disagreed, a tertiary consensus reading was derived by open discussion amongst the 3 arbiters (Fig 1). After each batch of 100 readings, two members of the arbitration panel provided verbal feedback to the primary readers on the basis of the disagreements identified and re-calibrated their interpretations by using examples of the 222 reference radiographs in the WHO library.

For the PCVIS project, the conclusions from the primary readers were used in cases where they agreed; where they did not agree, the result of the arbitration process was used[9]

### Analysis

Inter- and intra-observer agreements were estimated using two methods: percentage agreement and Cohen’s Kappa index. Kappa statistics were interpreted using a benchmark scale proposed by Altman; the strength of agreement was poor, fair, moderate, good or very good for Kappa statistics exceeding zero, 0.20, 0.40, 0.60, and 0.80, respectively[15]. Intra-observer agreement was estimated separately for each reader using the 5% of radiographs that were read twice. Agreement between reader A and reader B (i.e. inter-observer agreement) was estimated using the first reading of these pairs and their single reading of all other radiographs. We did not analyse agreement between radiologists providing arbitration because many of these were pooled decisions.

We estimated the sensitivity and specificity of the readings of Reader A against the arbitration panel results for all discordant readings, and for the sample of concordant readings; we then obtained a weighted summary estimate to take account of the fact that only a minority of the concordant images were reviewed by the arbitration panel. The overall sensitivity, s, was computed using the formula s = qs_agree_+(1-q) s_disagree,_ where s_agree_ is the sensitivity in the sample of concordant readings, s_disagree_ is the sensitivity in the sample of discordant readings, and q is an estimate of the probability of agreement between readers A and B when the radiograph is a true positive. The overall specificity was similarly defined, and we repeated the calculations of sensitivity and specificity for Reader B. Additional information on the calculations is outlined in S1 Appendix. Confidence intervals for these estimates were obtained using the delta method[16]. We used Stata version 11.2 for the analysis and Filemaker Pro for the database management.

### Ethical considerations

The study was approved by the KEMRI National Ethical Review Committee (SSC1049). Written informed consent was obtained from the parents/guardians of all the study participants.

## Results

A total of 2716 CXRs were digitized and read; 159 of these were read twice by each of the primary readers. The flow of radiographs through the stages of the study is shown in Fig 1. Of 2716 unique images, 2600 (96%) were designated adequate, 114 (4%) were designated sub-optimal by either of the primary readers and 2 were uninterpretable. Percent agreement between the primary readers on quality assessment scoring was high (96.0%) but the Cohen’s Kappa index was poor (0.15). Uninterpretable images were excluded from all further analyses. As the proportion of images that were sub-optimal was small and as the focus of the present study is on RCP which can still be derived from sub-optimal images, the remaining 2714 images were used for all remaining analyses.

In the final interpretations reported to the PCVIS project, 621 (23%) had consolidation, 78 (3%) had pleural effusion and 394 (15%) had Other Infiltrate; 1887 (70%) of the radiographs were classified as having no consolidation/infiltrate/effusion and 626 (23%) were classified as radiologically-confirmed pneumonia (RCP). Notably, 5 (<1%) RCP cases had pneumonia without consolidation.

The analysis of intra-observer agreement is shown in Table 2. Overall, Reader B was more consistent than Reader A. With a Kappa index of only 0.11 the repeatability of Reader A in identifying Other Infiltrate was poor. For the other two primary pathological classifications, the Kappa indices were moderate-to-good for Reader A (0.57–0.63) and good-to-very good for Reader B (0.71–0.85). Kappa indices for the composite conclusion, RCP, reflected these values.

**Table 2:** Intra-observer variability of the primary readers among a sub-sample of 159 images read twice by each reader.

	Reader A		Reader B	
End-point	Percent Agreement	Kappa	Percent Agreement	Kappa
Consolidation	86.8	0.59	90.6	0.71
Other Infiltrate	86.2	0.11	91.2	0.47
Pleural effusion	98.1	0.57	98.7	0.85
RCP*	88.7	0.63	93.1	0.78

*RCP is defined as images with consolidation or pleural fluid or both

The inter-observer variability analysis is shown in Table 3. Cohen’s Kappa indices were moderate-to-good for all pathological features except Other Infiltrate. For the composite conclusion, RCP, Kappa was 0.68.

**Table 3:** Inter-observer variability of the primary readers on 2714 images.

End-point	Percent Agreement	Kappa
Consolidation	86.0	0.61
Other Infiltrate	84.4	0.19
Pleural effusion	96.5	0.43
RCP*	89.1	0.68

*RCP is defined as images with consolidation or pleural fluid or both

**Fig 2:**
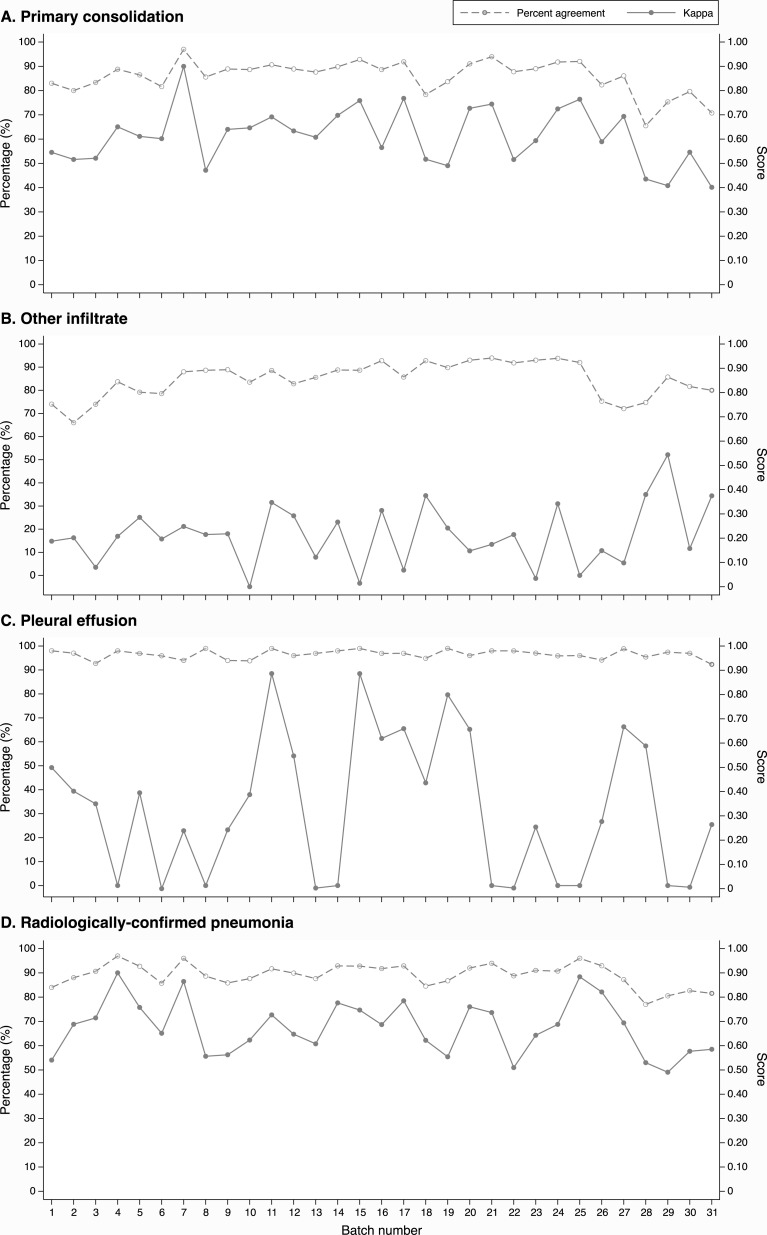
Inter-observer agreement between the two primary readers on pathological features and on radiologically-confirmed pneumonia against batch number.

To explore the effect of learning throughout the study, the inter-observer agreements for each of the radiological features and the final endpoint RCP are illustrated against sequential batch number (Fig 2). Linear regression of these trends are presented in S1 Table. There are no obvious trends in either percent agreement nor Cohen’s Kappa to suggest an improvement in inter-observer reliability over time.

Of the 1,767 images with concordant classifications from both primary readers, 232 (13%) were submitted to the arbitration panel as a quality control sample. The results of this analysis are shown in Table 4. The primary readers found consolidation in 68% (27/40) of the images in which the arbitration panel found consolidation and in none of the images in which the arbitration found no consolidation. Similar tables for the 949 images where the primary readers had discordant interpretations are shown in S2 Table.

**Table 4:** Performance of primary readers interpreting 232 chest radiographs selected at random from among their concordant readings compared against the final readings of the arbitration panel.

**A. Consolidation**				
	**Arbitration panel**			
**Primary readers**		Yes	No	Total
	Yes	27	0	27
	No	13	192	205
	Total	40	192	232
**B. Other Infiltrate**				
	**Arbitration panel**			
**Primary readers**		Yes	No	Total
	Yes	3	0	3
	No	37	192	229
	Total	40	192	232
**C. Pleural effusion**				
	**Arbitration panel**			
**Primary readers**		Yes	No	Total
	Yes	3	1	4
	No	5	223	228
	Total	8	224	232
**D. Radiologically-confirmed pneumonia (RCP)**				
	**Arbitration panel**			
**Primary readers**		Yes	No	Total
	Yes	27	0	27
	No	13	192	205
	Total	40	192	232

The sensitivity and specificity of the individual primary readers against the gold-standard of the arbitration panel conclusions is shown in Table 5. The sensitivity of the primary readers for RCP was 69% and 73% and the specificity was 96% and 95%.

**Table 5:** Weighted sensitivity and specificity of the primary readers’ conclusions against the arbiters’ gold-standard.

	Reader A		Reader B	
End-point	Sensitivity (95% CI)	Specificity (95% CI)	Sensitivity (95% CI)	Specificity (95% CI)
Consolidation	68.3 (61.8, 74.7)	96.4 (94.0, 98.9)	70.4 (64.0, 76.8)	95.4 (92.3, 97.9)
Other Infiltrate	20.3 (15.5, 25.1)	96.3 (93.9, 98.7)	29.5 (24.1, 35.0)	96.7 (94.3, 99.1)
Pleural effusion	36.8 (20.0, 54.6)	97.9 (92.5, 100)	49.5 (30.7, 67.4)	97.4 (91.9, 100)
RCP*	68.9 (62.6, 75.2)	96.3 (93.8, 98.7)	72.5 (66.2, 78.8)	95.2 (92.7, 97.7)

*RCP is defined as images with consolidation or pleural fluid or both

## Discussion

For readings of paediatric CXRs, following a WHO standard interpretation, this study shows that the intra- and inter-observer agreements were good for the detection of consolidation and also for the conclusion of radiologically-confirmed pneumonia. Against the gold-standard of the arbitration panel, the interpretations in the field by the primary readers were highly specific and moderately sensitive for RCP.

Among 159 images that underwent paired readings by the same primary readers, we found moderate to very good intra-observer agreement for the pathological features consolidation and pleural effusion, as well as for RCP as a final diagnosis. However, the results were appreciably better for one reader than for the other. The percentage self-agreement for RCP in our primary readers (89–93%) compares favourably with the range (76–99%) among the 20 readers in the original reliability study of the WHO standard[3]. The repeat images were presented at least 4 batches apart and, among 2716 original images, it is unlikely that the readers could recognise many images as repeats or remember their original designations. In contrast to the other features, Kappa values for Other Infiltrate were particularly low and this lack of intra-observer agreement is also reflected in the inter-observer variation study results.

The analysis of inter-observer reliability illustrated good agreement for radiologically-confirmed pneumonia with a Kappa index of 0.68 for the two primary readers. This is consistent with the results of the original reliability study of the WHO interpretive standard where the range of Kappa indices for 20 readers against the consensus reading was 0.21–0.81 with a median of 0.66[3]. We observed better agreement between observers for the conclusion RCP than for its constituent pathological findings, consolidation or pleural effusion. This suggests there were images where both readers identified pathology but where one labelled this as consolidation and the other labelled it as pleural effusion. Overall, inter-observer agreement on pleural effusion was only moderate and the Kappa index was considerably lower than in the intra-observer variation study. This difference may be accounted for by the fact that one study was undertaken on a subset of only 159 images, the other in all 2716, but it may also indicate that each reader had a conception of pleural effusion which was internally more consistent than their shared conception.

As with the intra-observer repeatability, the Kappa value for Other Infiltrate in the inter-observer repeatability study were poor (0.19). The original WHO reliability study did not examine inter-observer variation for Other Infiltrate in isolation but the Kappa values for the combination of abnormalities (Other Infiltrate or consolidation) among the 20 readers against the consensus value were 0.31–0.68 with a median of 0.59 which represents lower agreement than for RCP[3]. Other Infiltrate is a difficult concept upon which to focus agreement as it is defined more by exclusion than inclusion. According to the WHO standard[2] the reader examines the image for positive signs of RCP, i.e. consolidation or pleural effusion, and if there are none, he/she then makes an interpretation whether the radiograph is normal or contains an alternative abnormality in the lung fields. Vague opacities that are not sufficiently large or well defined to merit ‘consolidation’ may be interpreted as Other Infiltrate by some readers and as normal variants by others.

An alternative methodological explanation for the low Kappa indices obtained for Other Infiltrate, and also for pleural effusion, is given by their low prevalence. Cohen’s Kappa is known to yield paradoxically low results where the proportion of agreement expected by chance is high and the observed percent agreement is also high, which can occur at high or low trait prevalence[17]. In this study, the prevalence of pleural effusion was only 3% and Other Infiltrate was 15%. Several solutions have been advanced to overcome this limitation, one of which is Gwet’s AC1 statistic[18, 19]. We repeated the intra- and inter-observer analyses using AC1 and the results do illustrate higher indices for Other Infiltrate and pleural effusion than are observed with Cohen’s Kappa (see S3 and S4 Tables). However, the reliability objective of this study was to test whether the experience of the WHO standard in the PCVIS project deviated from that observed in the primary WHO reliability study, which confined its analysis of inter-observer variation to Kappa.

The table of sensitivities and specificities (Table 5) suggests that the primary readers had much higher thresholds for declaring radiological features than did the arbiters. This is reflected in very low numbers of ‘false positive’ identifications among true negative images but relatively high numbers of ‘false negatives’ among true positives: the specificities were high but the sensitivities only moderate. This suggests a form of observer bias which may be explained in one of two ways: either that the confidence to declare the presence of an abnormality grows with experience or, alternatively, that the presence of a higher level of review raises the threshold for declaration of radiological features. In the presence of peer-criticism a false positive is considered a greater error than a false negative.

In an ideal study design, the interpretations of the primary readers would be assessed against an independent biological gold-standard such as lung biopsy, post mortem or CT scans of chest[20, 21]. However, the validity objective of our study was to assess the effect of using local radiology readings on the outcome of our vaccine impact study. Given the high specificity of both readers for RCP, compared to the arbitration panel, the impact of the vaccine is likely to be measured as accurately, in relative terms (percentage reduction in disease, or vaccine effectiveness), as it would have been by the expert panel. The sensitivities observed, of 69% and 73%, suggest that the absolute impact of the vaccine preventable disease incidence [22] may be underestimated by approximately 30%.

One of the strengths in the design of this study was the programme of continuous training and re-calibration throughout the course of the study. However, we found no evidence that recurrent re-calibration improved the agreement between readers over time though it may have sustained the benefits of the WHO calibration training undertaken at the beginning of the study.

The quality of our images was generally good as we excluded only two (<1%). The PERCH multicentre study on pneumococcal aetiology, of which Kilifi was one of the sites, recorded an average of 10% (ranging from 4% to 20%) uninterpretable images across all sites[23]. Both our uninterpretable images were from the pre-vaccine period where quality control measures may not have been as stringent. The 4% sub-optimal images in this study would only have adversely affected the assessment of Other Infiltrate, which was not a focus of the present study.

After training on the WHO interpretive standard, the agreement between two readers on RCP was good, and repeatability was also good. Kappa indices for inter-observer variation on RCP were very similar to those in the original WHO reliability study. The sensitivity of the primary readers in the field, assessed against the gold-standard of an expert arbitration panel in Oxford, suggested that the local readers underestimated the occurrence of RCP by about 30% and this should be adjusted for in absolute analyses of vaccine impact, however, specificity was very high. Taken as a whole these results suggests that the use of local readers to define radiologically-confirmed pneumonia in a large study measuring the relative effectiveness of the pneumococcal conjugate vaccine is likely to produce an unbiased estimate of impact[9].

## Supporting information

S1 Appendix Methodology for estimating sensitivity and specificity. (PDF)

S1 Table Trend analysis: Linear regression of scores by batch. (PDF)

S2 Table Performance of primary readers in 947 radiographs with discordant readings by the primary readers compared against final readings of the arbitration panel. (PDF)

S3 Table Comparison of Gwet’s AC1 with percentage agreement and Cohen’s Kappa in the analysis of Intra-observer variation. (PDF)

S4 Table Comparison of Gwet’s AC1 with percent agreement and Cohen’s Kappa on Inter-observer variation of the primary readers on 2714 images. (PDF)
