# Supplementary material for: Reliability and validity of the World Health Organization reading standards for paediatric chest radiographs used in the field in an impact study of Pneumococcal Conjugate Vaccine in Kilifi, Kenya
Source: PLoS One. 2018 Jul 25;13(7):e0200715. doi: 10.1371/journal.pone.0200715 (PMC6059459; doi:10.1371/journal.pone.0200715)
Supplement: S1 Appendix — (PDF) [file pone.0200715.s001.pdf]

S1 Appendix. Methodology for estimating sensitivity and specificity

The study design provided two estimates of reader sensitivity: one from the 10% sample of radiographs reviewed when reader A and B were in agreement, and the other from radiographs reviewed when the two readers were in disagreement. To obtain an overall estimate of sensitivity we applied the law of total probability:

$$\mathrm{p}(T+ \mid D+) = \mathrm{p}(T+ \mid D+, A+)\mathrm{p}(A+ \mid D+) + \mathrm{p}(T+ \mid D+, A-)\,(1 - \mathrm{p}(A+ \mid D+))$$

Where

T+ = reader classifies radiograph pneumonia positive

D+ = radiograph true positive (as decided by the expert panel)

A+ = readers agree

A- = readers disagree.

To calculate probability of agreement for true positives we used data presented in Table 4, S2 Table, Figure 1, and the formula:

$$\mathrm{p}(A+ \mid D+) = \frac{\mathrm{p}(D+ \mid A+)\mathrm{p}(A+)}{\mathrm{p}(D+ \mid A+)\mathrm{p}(A+) + \mathrm{p}(D+ \mid A-)\mathrm{p}(A-)}$$

Similarly, to calculate specificity we used:

$$\mathrm{p}(T- \mid D-) = \mathrm{p}(T- \mid D-, A+)\mathrm{p}(A+ \mid D-) + \mathrm{p}(T- \mid D-, A-)\,(1 - \mathrm{p}(A+ \mid D-))$$

Where

T- = reader classifies radiograph pneumonia negative

D- = radiograph true negative.

And for the probability of agreement for true negatives we used:

$$\mathrm{p}(A+|D-)=\frac{\mathrm{p}(D-|A+)\mathrm{p}(A+)}{\mathrm{p}(D-|A+)\mathrm{p}(A+)+\mathrm{p}(D-|A-)\mathrm{p}(A-)}$$
