# Supplementary material for: Reliability and validity of the World Health Organization reading standards for paediatric chest radiographs used in the field in an impact study of Pneumococcal Conjugate Vaccine in Kilifi, Kenya
Source: PLoS One. 2018 Jul 25;13(7):e0200715. doi: 10.1371/journal.pone.0200715 (PMC6059459; doi:10.1371/journal.pone.0200715)
Supplement: S1 Table — (PDF) [file pone.0200715.s002.pdf]

S1 Table. Trend analysis: Linear regression of scores by batch

Percent agreement

**End-point**	**Slope***	**95% CI**	**P-value**
Consolidation	-0.23	-0.51 , 0.04	0.089
Other Infiltrate	-0.01	-0.09 , 0.07	0.841
Pleural effusion	0.20	-0.11 , 0.50	0.198
RCP	-0.17	-0.35 , 0.02	0.084

Kappa

**End-point**	**Slope***	**95% CI**	**P-value**
Consolidation	-0.22	-0.71 , 0.27	0.374
Other Infiltrate	-0.24	-1.48 , 0.99	0.690
Pleural effusion	0.23	-0.32 , 0.79	0.400
RCP	-0.24	-0.70 , 0.21	0.283

*The slope is measured as the change in percentage per batch. To analyse the Kappa scores they were first multiplied by 100.
