# Supplementary material for: Reliability and validity of the World Health Organization reading standards for paediatric chest radiographs used in the field in an impact study of Pneumococcal Conjugate Vaccine in Kilifi, Kenya
Source: PLoS One. 2018 Jul 25;13(7):e0200715. doi: 10.1371/journal.pone.0200715 (PMC6059459; doi:10.1371/journal.pone.0200715)
Supplement: S2 Table — (PDF) [file pone.0200715.s003.pdf]

S2 Table. Performance of primary readers in 947 radiographs with discordant readings by the primary readers compared against final readings of the arbitration panel

A. Consolidation

		Final arbitration		
		Yes	No	Total
**Primary reader A**	Yes	306	55	361
	No	139	447	586
**Primary reader B**	Yes	322	75	397
	No	123	427	550
Total		445	502	947

B. Other Infiltrate

		Final arbitration		
		Yes	No	Total
**Primary reader A**	Yes	116	60	176
	No	263	508	771
**Primary reader B**	Yes	179	53	232
	No	200	515	715
Total		379	568	947

C. Pleural effusion

		Final arbitration		
		Yes	No	Total
**Primary reader A**	Yes	22	32	54
	No	39	854	893
**Primary reader B**	Yes	37	44	81
	No	24	842	866
Total		61	886	947

D. Radiologically-confirmed pneumonia

		Final arbitration		
		Yes	No	Total
**Primary reader A**	Yes	321	58	379
	No	139	429	568
**Primary reader B**	Yes	349	78	427
	No	111	409	520
Total		460	487	947
