# Supplementary material for: Reliability and validity of the World Health Organization reading standards for paediatric chest radiographs used in the field in an impact study of Pneumococcal Conjugate Vaccine in Kilifi, Kenya
Source: PLoS One. 2018 Jul 25;13(7):e0200715. doi: 10.1371/journal.pone.0200715 (PMC6059459; doi:10.1371/journal.pone.0200715)
Supplement: S3 Table — (PDF) [file pone.0200715.s004.pdf]

S3 Table. Comparison of Gwet's AC1 with percentage agreement and Cohen's Kappa in the analysis of Intra-observer variation

End-point	Reader A			Reader B		
	Percent Agreement	Kappa	AC1	Percent Agreement	Kappa	AC1
Consolidation	86.8	0.59	0.83	90.6	0.71	0.88
Other Infiltrate	86.2	0.11	0.85	91.2	0.47	0.91
Pleural effusion	98.1	0.57	0.98	98.7	0.85	0.99
RCP*	88.7	0.63	0.84	93.1	0.78	0.90

*RCP is defined as images with consolidation or pleural fluid or both
