# Supplementary material for: Reliability and validity of the World Health Organization reading standards for paediatric chest radiographs used in the field in an impact study of Pneumococcal Conjugate Vaccine in Kilifi, Kenya
Source: PLoS One. 2018 Jul 25;13(7):e0200715. doi: 10.1371/journal.pone.0200715 (PMC6059459; doi:10.1371/journal.pone.0200715)
Supplement: S4 Table — (PDF) [file pone.0200715.s005.pdf]

S4 Table. Comparison of Gwet's AC1 with percent agreement and Cohen's Kappa on Inter-observer variation of the primary readers on 2714 images

End-point	Percent Agreement	Kappa	AC1
Consolidation	86.0	0.61	0.82
Other Infiltrate	84.4	0.19	0.83
Pleural effusion	96.5	0.43	0.96
RCP*	89.1	0.68	0.84

*RCP is defined as images with consolidation or pleural fluid or both
